# Exercise-based interventions for depression in women with polycystic ovary syndrome: a systematic review and meta-analysis

**DOI:** 10.3389/fpubh.2026.1802184

**Published:** 2026-04-13

**Authors:** Yi Zheng, Dong-Qiang Zhang

**Affiliations:** 1 Capital University of Physical Education and Sports, Martial Arts and Performance Academy, Beijing, China; 2 Physical Education Department, Xiamen University Tan Kah Kee College, Zhangzhou, Fujian, China

**Keywords:** depression, exercise, meta-analysis, polycystic ovary syndrome, randomised controlled trials

## Abstract

**Background:**

Depressive symptoms are highly prevalent among women with polycystic ovary syndrome (PCOS), with previous studies suggesting a substantially elevated burden of depression and depressive symptoms in this population compared with women without PCOS, exacerbating the metabolic and reproductive burden of the disorder. Although exercise is recommended as a core component of PCOS management, its specific effects on depressive outcomes in this population remain unclear because evidence from randomised controlled trials has not been comprehensively and quantitatively synthesised.

**Objective:**

To evaluate the efficacy of exercise interventions in reducing depressive symptoms among women with PCOS and to explore whether intervention characteristics may moderate treatment effects.

**Methods:**

A systematic review and meta-analysis of randomised controlled trials was conducted. Standardised mean differences (SMDs) with 95% confidence intervals (CIs) were calculated using a random- or fixed-effects model as appropriate. Pre-specified subgroup analyses examined exercise type, intervention format, weekly frequency, and programme duration. But were considered exploratory because of the limited number of included studies, Sensitivity analyses and publication bias assessments were performed.

**Results:**

Seven trials met the inclusion criteria. Exercise interventions significantly reduced depressive symptoms compared with controls (SMD = −0.75, 95% CI −0.96 to −0.55). In exploratory subgroup analyses, resistance exercise and interventions with higher weekly frequency or longer duration showed numerically larger effect estimates; however, between-subgroup differences were not statistically significant. Sensitivity analyses showed that the pooled effect estimate was not materially altered by sequential study removal, and no significant publication bias was detected.

**Conclusion:**

Structured exercise may be a promising non-pharmacological strategy for reducing depressive symptoms in women with PCOS. However, the findings should be interpreted cautiously in light of the small number of trials and limited sample sizes. Further high-quality trials are required to determine optimal exercise prescriptions and long-term psychological benefits.

**Systematic review registration:**

The protocol was registered on https://www.crd.york.ac.uk/PROSPERO/view/CRD42022375480 (CRD420261285288).

## 1 Background

Depression is one of the most prevalent mental disorders worldwide, affecting an estimated 280 million people and representing a major contributor to global disability. Beyond its substantial individual burden, depression imposes considerable societal and economic costs (1, 2). Although pharmacotherapy and psychotherapy are established treatments, under-recognition, limited accessibility, concerns regarding adverse effects and long-term adherence highlight the ongoing need for scalable, sustainable non-pharmacological strategies (3–5).

Polycystic ovary syndrome (PCOS) is one of the most common endocrine–metabolic disorders in women of reproductive age, with a reported prevalence of approximately 6–13% depending on the diagnostic criteria applied (6, 7). It is defined by ovulatory dysfunction, hyperandrogenism and polycystic ovarian morphology, and is frequently accompanied by insulin resistance, obesity and elevated cardiometabolic risk (8). Increasing evidence indicates that the burden of PCOS extends beyond reproductive and metabolic dysfunction to encompass substantial psychological morbidity. Phenotypic features such as weight gain, acne and hirsutism, together with fertility-related stress and the chronicity of metabolic disturbance, may contribute to heightened vulnerability to depressive symptoms (9). Epidemiological studies consistently report a significantly increased risk of depression among women with PCOS compared with those without the condition, underscoring the necessity of integrating mental health considerations into PCOS management (10). In addition, contemporary international PCOS guidelines identify lifestyle intervention, including regular physical activity and structured exercise, as a core component of first-line management, given its potential benefits for metabolic, reproductive and psychological health (11, 12).

Exercise has emerged as a promising strategy for the prevention and treatment of depression (13). Its putative mechanisms include modulation of systemic inflammation, improvements in insulin sensitivity, regulation of neurotransmitters and neurotrophic factors, and normalisation of hypothalamic–pituitary–adrenal (HPA) axis activity (14). In the context of PCOS, physical activity confers additional metabolic benefits, including reductions in adiposity and improvements in insulin resistance and cardiometabolic parameters. Previous studies and guideline-based recommendations have therefore highlighted exercise and lifestyle modification as important elements of PCOS care (12, 15). These physiological and psychological effects suggest that exercise may represent a multidimensional intervention for addressing depressive symptoms in this population (16, 17). However, much of the proposed mechanistic rationale is derived from broader literature on depression and exercise or from studies focused primarily on metabolic outcomes in PCOS, rather than from trials directly designed to examine psychological mechanisms in women with PCOS.

However, evidence regarding the efficacy of exercise for depressive symptoms in women with PCOS remains limited in volume and not yet quantitatively synthesised in a sufficiently focused manner across randomised controlled trials (18–21). Variability in exercise modality (aerobic versus resistance training), frequency, duration and delivery format (individual versus group-based) may contribute to heterogeneity in reported outcomes. Moreover, differences in depression assessment tools and study designs limit the ability to draw definitive conclusions regarding overall effectiveness and potential moderators of treatment response. Existing literature has also tended to emphasise metabolic or reproductive endpoints, with depressive symptoms often treated as secondary outcomes, further limiting clarity regarding the mental health effects of exercise in this population. To address these gaps, the present study systematically identified and synthesised evidence from randomised controlled trials, applying meta-analytic methods to quantify the overall impact of exercise interventions on depressive symptoms in women with PCOS. Pre-specified subgroup analyses were conducted to examine whether variations in intervention characteristics influenced effect magnitude. By integrating available trial evidence, this study aims to provide a more focused quantitative summary of the available evidence for the mental health management of PCOS.

## 2 Meta-analysis data

This meta-analysis was performed according to the Preferred Reporting Items for Systematic Reviews and Meta-Analysis statement and the Cochrane Collaboration Handbook. The protocol was registered on PROSPERO (CRD420261285288).

### 2.1 Data sources and searches

The Meta-analysis search was conducted by two independent reviewers (ZY and ZD-Q) in four databases: the Cochrane Library, Embase, PubMed and Web of Science, and was designed to retrieve articles up to Jan 2026, with disagreements resolved by consensus and by a third reviewer (ZXD) in case of disagreement. Terms from the Medical Subject Headings (MeSH) and words from the text were used as follows: (“Polycystic Ovary Syndrome” OR “Ovary Syndrome, Polycystic” OR “Syndrome, Polycystic Ovary” OR “Polycystic Ovarian Syndrome” OR “Ovarian Syndrome, Polycystic” OR “Polycystic Ovary Syndrome 1” OR “Sclerocystic Ovarian Degeneration” OR “Sclerocystic Ovary Syndrome” OR “Sclerocystic Ovary Syndrome” OR “Stein-Leventhal Syndrome” OR “Stein Leventhal Syndrome” OR “Syndrome, Stein-Leventhal” OR “Sclerocystic Ovaries” OR “Sclerocystic Ovary”) AND (“Depression” OR “Depressive Symptoms” OR “Depressive Symptom” OR “Symptom, Depressive” OR “Emotional Depression” OR “Depression, Emotional”) AND (“Exercise” OR “Exercises” OR “Physical Activity” OR “Activities, Physical” OR “Activity, Physical” OR “Physical Activities” OR “Exercise, Physical” OR “Exercises, Physical” OR “Physical Exercise” OR “Physical Exercises” OR “Acute Exercise” OR “Acute Exercises” OR “Exercise, Acute” OR “Exercises, Acute” OR “Exercise, Isometric” OR “Exercises, Isometric” OR “Isometric Exercises” OR “Isometric Exercise” OR “Exercise, Aerobic” OR “Aerobic Exercise” OR “Aerobic Exercises” OR “Exercises, Aerobic” OR “Exercise Training” OR “Exercise Trainings” OR “Training, Exercise” OR “Trainings, Exercise”) Specific details of the search algorithms for each database are provided in Supplementary material 1. Where full texts were not readily accessible or where key outcome data were incomplete, attempts were made to contact the corresponding authors for clarification where feasible.

### 2.2 Inclusion and exclusion

Eligibility criteria were defined *a priori* according to the PICOS (Population, Intervention, Comparison, Outcomes, Study design) framework. Population (P): We included studies enrolling women diagnosed with polycystic ovary syndrome (PCOS) according to established diagnostic criteria, including the Rotterdam criteria, the National Institutes of Health (NIH) criteria, or the Androgen Excess and PCOS Society criteria. Participants were required to be adolescents or adult women of reproductive age; Intervention (I): Eligible interventions comprised structured exercise programmes as the primary therapeutic component. For the purposes of this review, a structured exercise programme was defined as a planned, repetitive, and purposive programme of physical training with prespecified modality, frequency, intensity and/or duration. Exercise modalities included, but were not limited to, aerobic training (e.g., walking, running, cycling), high-intensity interval training (HIIT), resistance training, and combined aerobic–resistance training. Interventions were required to provide sufficient detail regarding exercise modality, intensity, frequency and duration. Multicomponent lifestyle programmes were included only if the effect of exercise could be isolated or clearly attributed, Studies that only provided general lifestyle advice, non-specific physical activity encouragement, or health education without a clearly prescribed exercise protocol were excluded. Comparison (C): Eligible comparators included usual care, no intervention, wait-list control, or non-exercise interventions (e.g., health education). Studies were required to report comparative data between intervention and control groups; Outcomes (O): The primary outcome was change in depressive symptoms, assessed using validated and standardised scales, such as the Beck Depression Inventory (BDI), the Hospital Anxiety and Depression Scale (HADS), the Center for Epidemiologic Studies Depression Scale (CES-D), or equivalent instruments. Studies were required to report sufficient quantitative data (e.g., means and standard deviations at baseline and post-intervention, or change scores) to permit calculation of effect sizes; Study design (S): Only randomised controlled trials (RCTs) were eligible for inclusion. Observational studies, non-randomised trials, case reports, conference abstracts and review articles were excluded.

Studies were excluded if they met any of the following criteria:

(1) did not include women diagnosed with PCOS;
(2) did not involve exercise as the primary intervention;
(3) lacked a control group;
(4) did not report depression-related outcomes using validated instruments;
(5) insufficient data were available to calculate effect sizes;
(6) were non-randomised studies, reviews, abstracts, or duplicate publications.

For trials with multiple eligible exercise arms sharing a common control group, relevant intervention arms were combined where conceptually appropriate to avoid double-counting of the control group. When combination was not appropriate, the shared control group was divided proportionally in accordance with Cochrane recommendations.

### 2.3 Assessment of risks of bias

In accordance with the Cochrane Collaboration guidelines, reviewers ZY and ZD-Q independently conducted a structured risk-of-bias assessment for each included study. The Cochrane Risk of Bias tool version 1 (RoB 1) was used to assess the following domains: random sequence generation, allocation concealment, blinding of participants and personnel, blinding of outcome assessment, incomplete outcome data, selective reporting, and other potential sources of bias. All assessments were performed independently, and disagreements were resolved through discussion or consultation with a third reviewer (ZXD) when necessary. The detailed results of these evaluations are summarised in the Risk of Bias Table (Supplementary material 2).

In interpreting the pooled effects of exercise interventions on depressive symptoms in women with PCOS, risk-of-bias considerations were explicitly incorporated into the analytical framework. Risk-of-bias judgments were considered during interpretation of the pooled findings, particularly when discussing the certainty and potential limitations of the evidence.

### 2.4 Data extraction

Using a pre-specified and standardised data extraction form, two reviewers (ZY and ZD-Q) independently extracted all relevant information from each included study. The use of a uniform template was intended to enhance consistency, minimise extraction errors, and ensure the integrity of the analytical dataset. Any discrepancies were resolved through discussion and, when necessary, adjudicated by a third reviewer (ZXD) to achieve consensus.

### 2.5 Assessment of overall effect size

Statistical analyses were performed using Review Manager (RevMan, version 5.3). Effect sizes were derived from standardised depression assessment scales across the included trials. For each study, Hedges’ g standardised mean differences (SMDs) were calculated to quantify intervention effects, with values of 0.2, 0.4 and 0.8 conventionally interpreted as small, medium, and large, respectively. All effect sizes were coded such that negative values reflected improvement in depressive symptoms, and statistical significance was defined as *p* < 0.05.

Because the included trials involving women with polycystic ovary syndrome (PCOS) used different validated measures of depressive symptoms, SMDs were employed to permit synthesis across heterogeneous scales. SMDs and their 95% confidence intervals (CIs) were calculated using the Practical Meta-Analysis Effect Size Calculator. Between-study heterogeneity was assessed using the Q statistic and the *I*^2^ index, with thresholds of 25, 50, and 75% representing low, moderate, and high heterogeneity, respectively. A random-effects model was applied when heterogeneity was moderate to high; otherwise, a fixed-effects model was used. Where both baseline and post-intervention data were available, effect sizes were derived using comparable outcome metrics across studies to maximise consistency of pooling.

### 2.6 Subgroup analysis of exercise intervention programmes

Pre-specified subgroup analyses were conducted to explore potential sources of between-study heterogeneity and to examine whether intervention characteristics moderated the effect of exercise on depressive symptoms in women with PCOS. Subgroups were defined according to four key dimensions of exercise prescription. First, interventions were categorised by type of exercise, distinguishing aerobic exercise from resistance exercise. Studies involving combined modalities were classified according to the predominant training component, as defined by the authors; Second, interventions were stratified by delivery format, comparing individually delivered programmes with group-based exercise interventions, to assess whether social interaction and structured supervision influenced treatment effects; Third, subgroup analyses were performed based on weekly training frequency, comparing programmes conducted three times per week with those conducted five times per week;

Fourth, interventions were classified according to programme duration, dichotomised as ≤16 weeks or >16 weeks, to evaluate whether longer exposure was associated with differential effects on depressive outcomes.

Subgroup differences were examined using the test for interaction implemented in RevMan. Given the small number of included trials and the limited number of studies within several subgroup categories, these analyses were considered exploratory and were interpreted cautiously. No inferences regarding the superiority of one subgroup over another were made in the absence of statistically significant between-subgroup interaction tests.

## 3 Results

### 3.1 Search process

**Figure 1:**
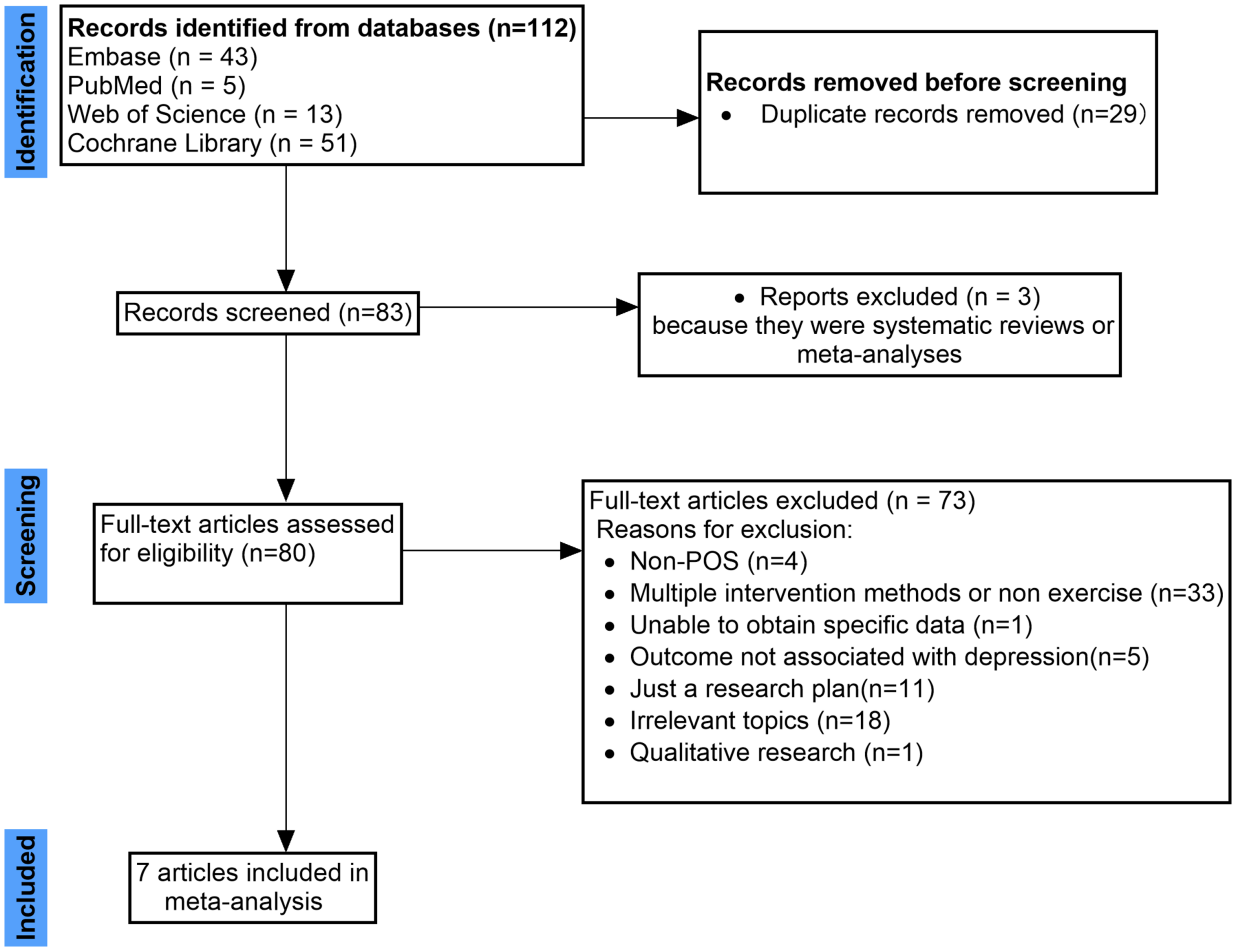
Flowchart and selection of studies.

The literature search identified 112 records from the four electronic databases. After removal of 29 duplicate records, 83 articles remained for title and abstract screening. Of these, three records were excluded at this stage because they were systematic reviews or meta-analyses, leaving 80 full-text articles assessed for eligibility. Seventy-three full-text articles were excluded for the following reasons: participants were not diagnosed with PCOS (*n* = 4); interventions involved multiple components or did not primarily consist of exercise (*n* = 33); specific data required for analysis were unavailable (*n* = 1); outcomes were not related to depressive symptoms (*n* = 5); the article represented a research protocol only (*n* = 11); topics were not relevant to the review question (*n* = 18); or the study employed a qualitative design (*n* = 1). Ultimately, seven randomised controlled trials met the eligibility criteria and were included in the meta-analysis. The study selection process is summarised in Figure 1. The relatively small number of records retrieved from the electronic databases may be due to the narrow scope of the review and the strict eligibility criteria, as this study specifically focused on exercise-based interventions for depressive symptoms in women with polycystic ovary syndrome. Where available, the number of records retrieved from each database is shown in the PRISMA flow diagram.

### 3.2 Characteristics of the included studies and participants

The characteristics of the seven studies included in this meta-analysis are summarised in Table 1. Detailed information is provided on sample size, mean participant age, intervention and control group design, type of exercise intervention, intervention duration and frequency, as well as the depression outcome measures used in each study. Overall, the included trials varied in exercise modality, delivery format, training frequency, programme duration, and depression assessment tools, which may have contributed to between-study variation in effect estimates. The total number of participants across all included studies was *n* = 346.

**Table 1:** Characteristics of the included studies and participants.

Studies	Sample size (IG/CG)	Age range (IG/CG)	IG Type	Frequency/Duration	Outcome measures	Country	Exercise format
Thomson, R. L 2016	11:13		Aerobic Exercise	5 times weekly/20 weeks	CES-D	Australia	Individual
Kogure, G. S (1) 2020	37:38	29.1 ± 5.3/28.5 ± 5.8	Aerobic Exercise	3 times weekly/16 weeks	HADS	Brazil	Individual
Kogure, G. S (2) 2020	35:38	29.0 ± 4.3/28.5 ± 5.8	Aerobic Exercise	3 times weekly/16 weeks	HADS	Brazil	Individual
Lopes, I P (1) 2018	23:24	30.2 ± 5.1/28.8 ± 6.0	Aerobic Exercise	3 times weekly/16 weeks	HADS	Brazil	Individual
Lopes, I P (2) 2018	22:24	29.4 ± 4.1/28.8 ± 6.0	Aerobic Exercise	3 times weekly/16 weeks	HADS	Brazil	Individual
Santos, I. K 2022	12:11	26.60 ± 3.92/26.6 ± 4.68	HIIT	12 weeks	DASS-21	Brazil	Group
Stener–Victorin, E 2013	29:15	29.9 ± 4.4	Aerobic Exercise	3 times weekly/16 weeks	MADRS-S	Sweden	Individual
Thomson, R. L (1) 2010	15:14	29.3 ± 0.7	Aerobic Exercise	5 times weekly/20 weeks	CES-D	Australia	Individual
Thomson, R. L (2) 2010	20:14	29.3 ± 0.7	combined resistance	5 times weekly/20 weeks	CES-D	Australia	Individual
Vizza, L. 2016	7:6	26 ± 7/29 ± 3	resistance training	3 times weekly/12 weeks	DASS-21	Australia	Group

IG = intervention group; CG = control group; DASS-21 = depression anxiety and stress scale; CES-D=centre for epidemiologic studies depression scale; HADS = hospital anxiety and depression scale; HIIT = high-intensity interval training; MADRS-S = Montgomery–Asberg depression rating scale.

### 3.3 Risks of bias

The risk of bias assessment for the seven included randomised controlled trials is summarised in Supplementary material 2. Overall, the methodological quality of the included studies was mixed, with several domains reflecting inherent challenges in exercise-based behavioural interventions.

All seven trials were judged to be at low risk of bias for random sequence generation, indicating appropriate randomisation procedures. With respect to allocation concealment, five studies were assessed as having a low risk of bias, while the remaining two studies provided insufficient information and were therefore rated as having an unclear risk. Blinding posed a notable limitation. For blinding of participants and personnel, six studies were judged to be at high risk of bias, reflecting the practical difficulty of masking exercise interventions, while one study was assessed as low risk. Similarly, for blinding of outcome assessment, two studies were rated as high risk of bias and five as unclear, owing to limited reporting on assessor blinding procedures. All included trials were judged to be at low risk of bias for incomplete outcome data, suggesting adequate handling of attrition and missing data. Regarding selective reporting, five studies were assessed as low risk, whereas two were rated as unclear due to the absence of accessible study protocols or pre-specified outcomes.

For other sources of bias, six studies were judged to be at unclear risk, primarily because of insufficient reporting on potential methodological or contextual confounders, while one study was assessed as having a low risk of bias. Taken together, these findings indicate that while randomisation and outcome completeness were generally robust across studies, limitations related to blinding and reporting transparency may have influenced the pooled estimates and should be considered when interpreting the findings.

### 3.4 Meta-analysis

#### 3.4.1 Baseline period test

A pooled analysis of baseline depressive symptom scores was conducted to assess comparability between intervention and control groups prior to the implementation of exercise programmes. Across the included comparisons, the aggregated effect size showed no significant difference at baseline [(22) SMD = −0.085, 95% CI −0.351 to 0.181, *p* = 0.532]. Between-study heterogeneity was negligible (*χ*^2^ = 0.95, d.f. = 9, *p* = 1.000), with an estimated between-study variance of *τ*^2^ = 0.000, indicating a high degree of consistency across trials.

**Figure 2:**
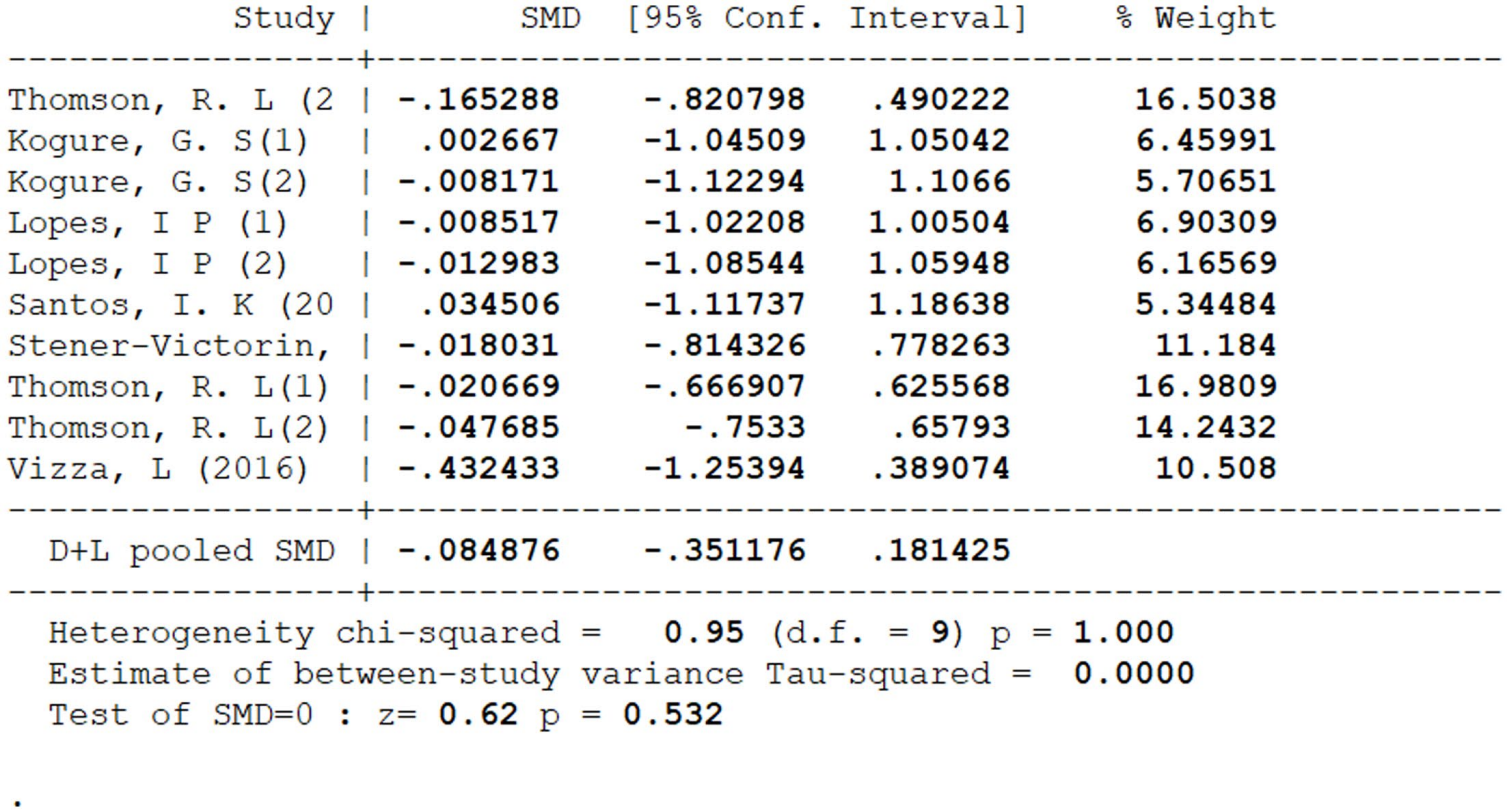
Baseline period test.

These results, presented in detail in Figure 2, indicate that intervention and control groups were well balanced in depressive symptom severity at study entry. The absence of significant baseline differences supports the comparability of groups before intervention, providing context for subsequent analyses of post-intervention effects.

#### 3.4.2 Meta-analysis result

**Figure 3:**
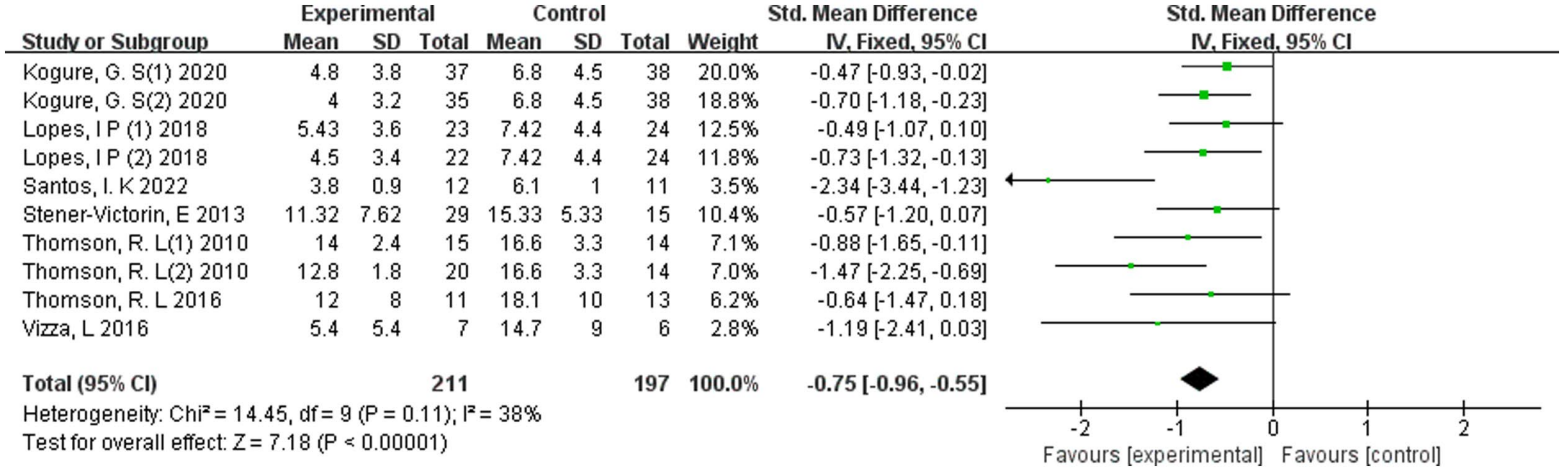
Forest plot of exercise for depression in PCOS.

The pooled analysis included seven trials involving 346 participants and demonstrated a statistically significant beneficial effect of exercise interventions on depressive symptoms in women with PCOS. As shown in Figure 3, the overall standardised mean difference indicated a moderate-to-large reduction in depressive symptoms favouring the exercise groups compared with controls (SMD = −0.75, 95% CI −0.96 to −0.55; *p* < 0.00001). Between-study heterogeneity was low to moderate, suggesting acceptable consistency across the included trials. The direction of effect was largely uniform, with the majority of studies demonstrating reductions in depressive symptom scores following exercise interventions.

Collectively, these findings suggest that structured exercise may be associated with improvement in depressive symptoms among women with PCOS. However, the relatively small number of included trials and limited sample sizes should be considered when interpreting the magnitude and certainty of the pooled effect. Detailed forest plot results are presented in Figure 3.

To assess the robustness of the pooled estimate, a leave-one-out sensitivity analysis was conducted, with each trial sequentially removed from the meta-analysis. The overall effect size remained materially unchanged, and heterogeneity indices exhibited no meaningful fluctuations, indicating that the aggregated findings were not unduly influenced by any single study. These results support the relative consistency of the pooled estimate, although they do not eliminate uncertainty related to the small evidence base.

#### 3.4.3 Publication bias test

**Figure 4:**
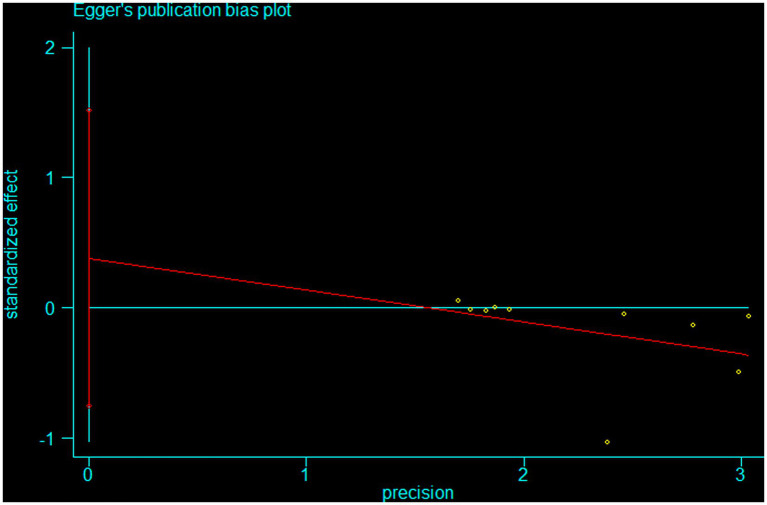
Egger’s publication bias plot.

**Figure 5:**
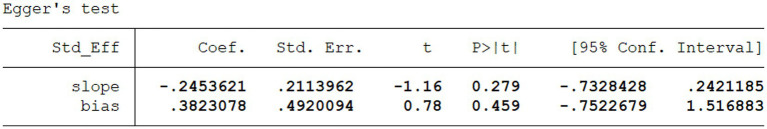
Egger’s test.

Potential publication bias was assessed visually using a funnel plot and statistically using Egger’s regression test. The funnel plot (Figure 4) appeared broadly symmetrical, with effect estimates distributed evenly around the pooled estimate across varying levels of precision, suggesting no marked small-study effects. Egger’s regression analysis further indicated no statistically significant evidence of publication bias. The intercept (bias) term was non-significant (*β* = 0.38, SE = 0.49, *p* = 0.459), and the regression slope was likewise non-significant (*p* = 0.279), with confidence intervals crossing zero (Figure 5).

Taken together, the funnel plot and Egger test did not suggest clear evidence of publication bias in this meta-analysis. However, these findings should be interpreted cautiously because the small number of included studies limits the power of formal publication bias tests.

#### 3.4.4 Subgroup analysis

Pre-specified subgroup analyses were undertaken to examine whether key characteristics of exercise prescription and programme delivery influenced the magnitude of treatment effects on depressive symptoms in women with PCOS. Interventions were stratified according to four dimensions.

First, analyses were conducted by type of exercise, comparing aerobic interventions with resistance-based programmes. Second, studies were categorised by delivery format, distinguishing individually delivered exercise from group-based programmes. Third, subgroup comparisons were performed according to weekly training frequency, contrasting interventions delivered three times per week with those conducted five times per week. Finally, programmes were stratified by duration, dichotomised as ≤16 weeks and >16 weeks.

Across these subgroup comparisons, effect estimates were examined descriptively together with formal tests for subgroup differences. Given the limited number of studies and participants within several subgroup categories, these analyses were considered exploratory and were interpreted cautiously. Accordingly, they should not be taken as definitive evidence regarding the superiority of one intervention characteristic over another.

##### 3.4.4.1 Intervention format

**Figure 6:**
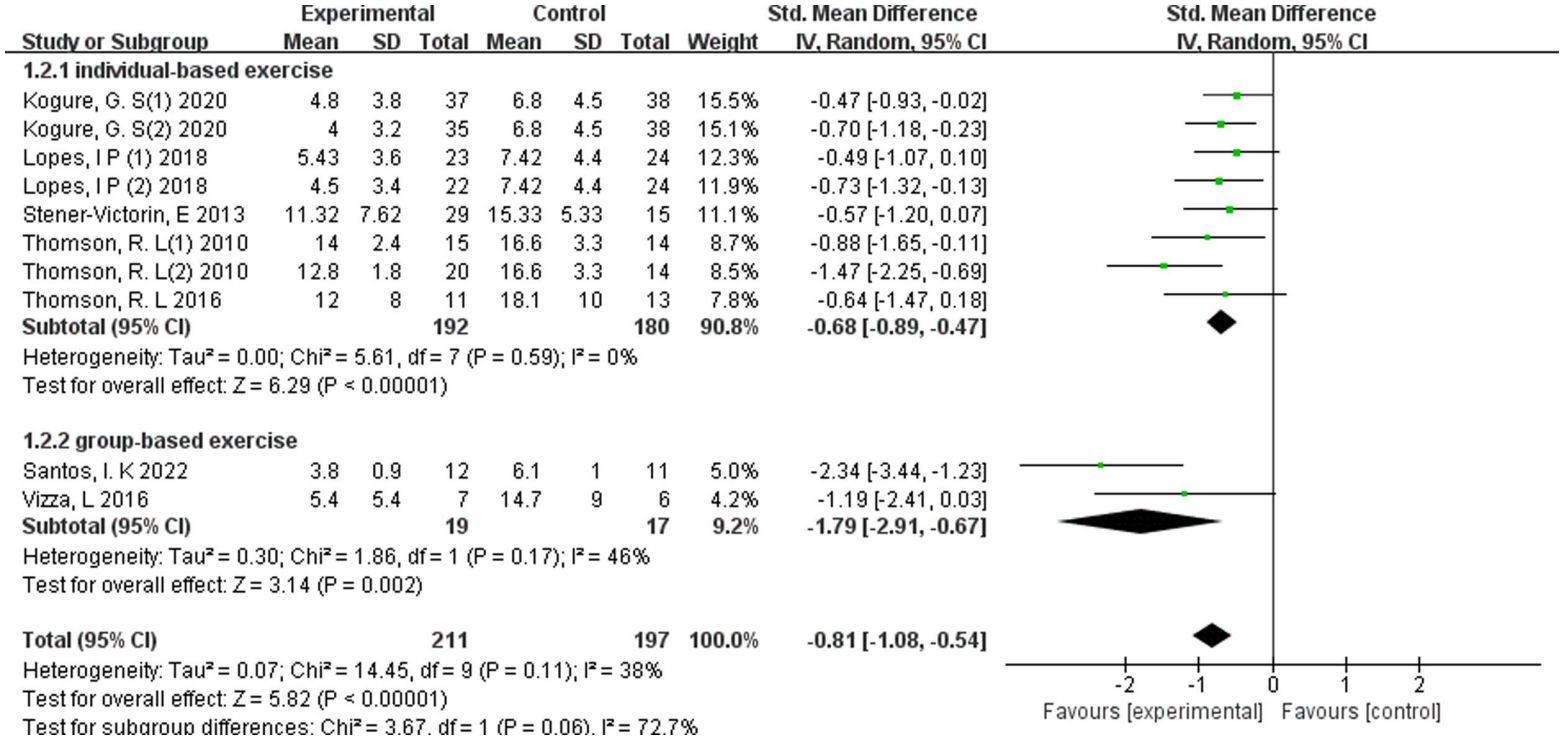
Forest plot of subgroup analyses by individual versus group.

Subgroup analysis according to intervention format revealed that both individually delivered and group-based exercise programmes were associated with significant reductions in depressive symptoms among women with PCOS (Figure 6).

For individually delivered interventions (*k* = 5 studies, *n* = 296 participants), the pooled estimate indicated a moderate and statistically significant effect (SMD = −0.68, 95% CI − 0.89 to −0.47; Z = 6.29, *p* < 0.00001), with no evidence of between-study heterogeneity (*I*^2^ = 0%). In contrast, group-based programmes (*k* = 2 studies, *n* = 36 participants) demonstrated a larger pooled effect size (SMD = −1.79, 95% CI −2.91 to −0.67; *Z* = 3.14, *p* = 0.002), although heterogeneity was moderate (*I*^2^ = 46%). The magnitude of effect was numerically greater in group-based interventions compared with individually delivered programmes. However, the test for subgroup differences did not reach conventional levels of statistical significance (*p* = 0.06; *I*^2^ for subgroup difference = 72.7%), indicating that while group-based exercise appeared to confer a stronger effect, this superiority did not meet statistical criteria for a definitive between-group difference.

These findings suggest that exercise interventions were associated with reduced depressive symptoms in both delivery formats. Although the pooled effect estimate was numerically larger for group-based programmes, the between-subgroup difference was not statistically significant, and this exploratory comparison should be interpreted cautiously.

##### 3.4.4.2 Exercise types

**Figure 7:**
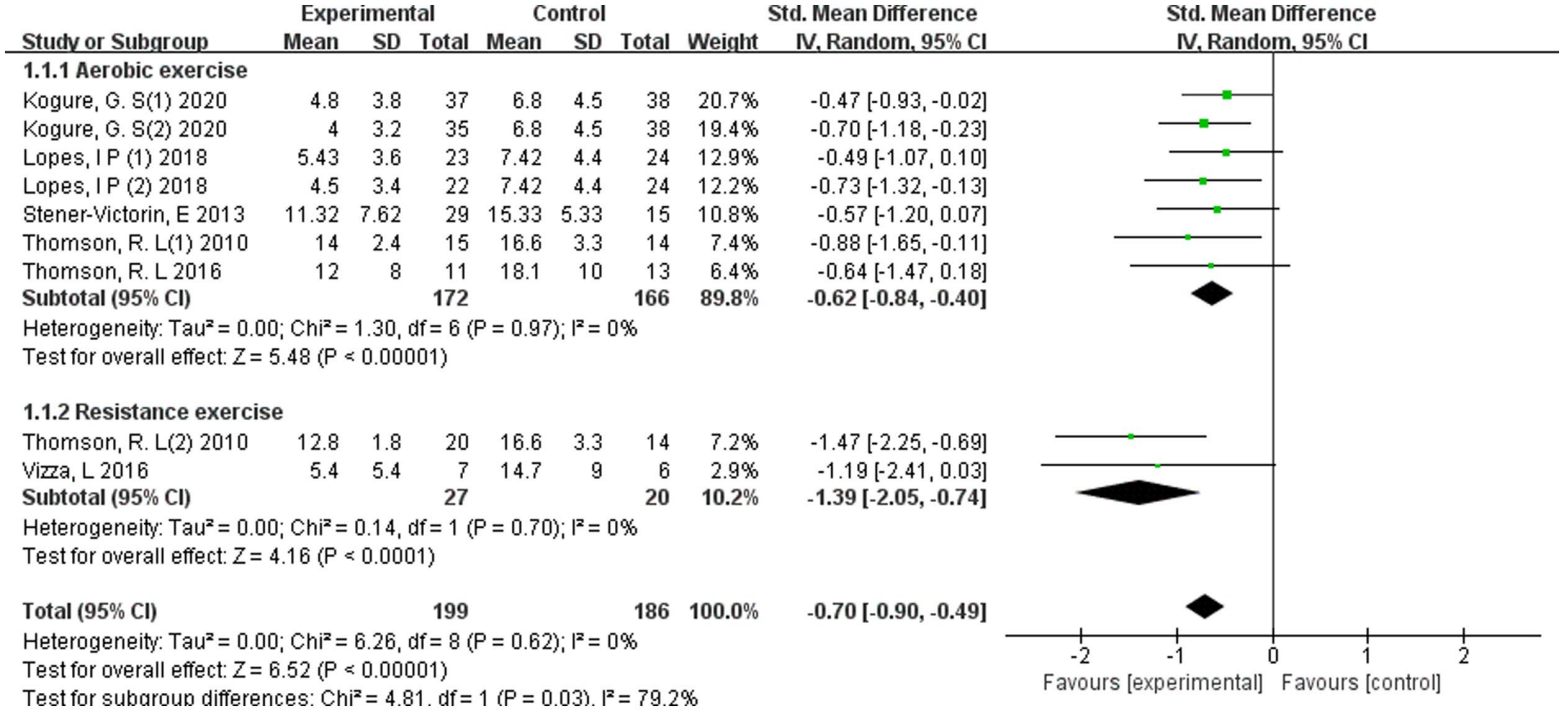
Forest plot of subgroup analyses by type of exercise.

Subgroup analysis stratified by exercise modality demonstrated that both aerobic and resistance-based interventions were associated with significant reductions in depressive symptoms among women with PCOS (Figure 7).

For aerobic exercise (*k* = 5 studies, *n* = 276 participants), the pooled effect indicated a moderate and statistically significant improvement compared with controls (SMD = −0.62, 95% CI − 0.84 to −0.40; *p* < 0.00001), with no evidence of heterogeneity (*I*^2^ = 0%). Resistance-based interventions (*k* = 2 studies, *n* = 47 participants) yielded a larger pooled effect size (SMD = −1.39, 95% CI −2.05 to −0.74; *p* < 0.0001), likewise with negligible heterogeneity (*I*^2^ = 0%).

The magnitude of effect was significantly greater for resistance exercise compared with aerobic exercise, and the test for subgroup differences confirmed a statistically significant between-group difference (*p* = 0.03; *I*^2^ for subgroup difference = 79.2%). Within the limitations of the small number of included studies, this exploratory finding suggests that resistance-based interventions may be associated with larger reductions in depressive symptoms than aerobic interventions. However, this result should be interpreted cautiously and requires confirmation in larger comparative trials.

##### 3.4.4.3 Training frequency

**Figure 8:**
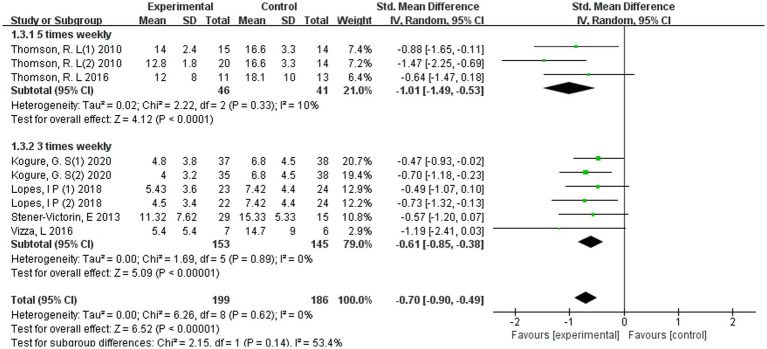
Forest plot of subgroup analyses by training frequency.

Subgroup analysis according to weekly training frequency indicated that both three-times-per-week and five-times-per-week exercise programmes were associated with significant reductions in depressive symptoms in women with PCOS (Figure 8).

Interventions delivered five times weekly (*k* = 2 studies, *n* = 73 participants) demonstrated a pooled effect size of SMD = −1.01 (95% CI − 1.49 to −0.53; *p* < 0.0001), with low heterogeneity (*I*^2^ = 10%). Programmes conducted three times per week (*k* = 3 studies, *n* = 2,396 participants) yielded a moderate and statistically significant effect (SMD = −0.61, 95% CI −0.85 to −0.38; *p* < 0.00001), with no observed heterogeneity (*I*^2^ = 0%).

Although the magnitude of effect was numerically greater in the five-times-per-week subgroup compared with the three-times-per-week subgroup, the test for subgroup differences was not statistically significant (*p* = 0.14; *I*^2^ for subgroup difference = 53.4%). Accordingly, these exploratory findings do not support a definitive conclusion that higher weekly training frequency is superior.

##### 3.4.4.4 Intervention duration

**Figure 9:**
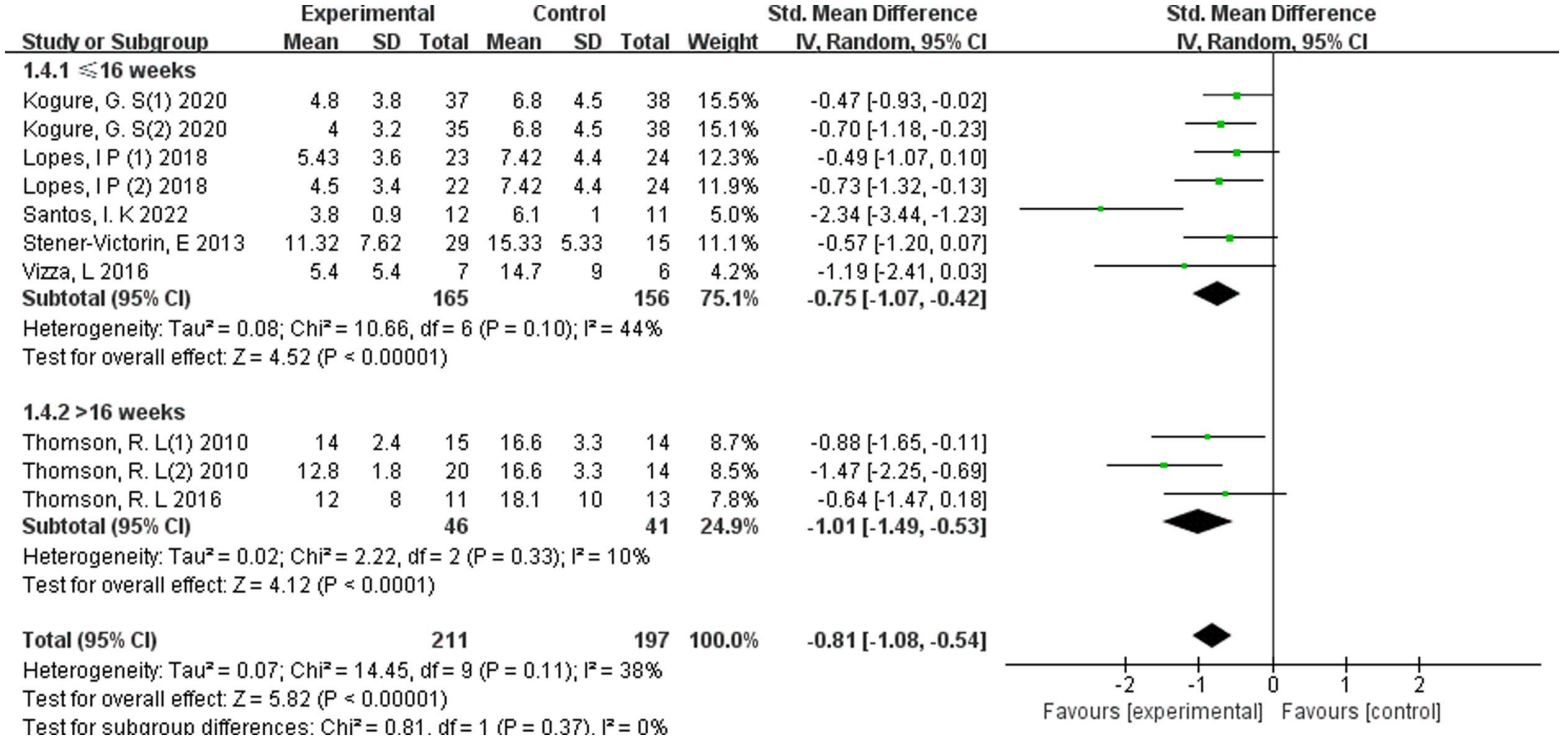
Forest plot of subgroup analyses by duration of the exercise programmes.

Subgroup analysis stratified by programme duration indicated that both shorter (≤16 weeks) and longer (>16 weeks) exercise interventions were associated with significant reductions in depressive symptoms among women with PCOS (Figure 9).

Programmes lasting ≤16 weeks (*k* = 5 studies, *n* = 258 participants) demonstrated a pooled effect size of SMD = −0.75 (95% CI −1.07 to −0.42; *p* < 0.00001), with moderate heterogeneity (*I*^2^ = 44%). Interventions extending beyond 16 weeks (*k* = 2 studies, *n* = 73 participants) yielded a larger pooled effect (SMD = −1.01, 95% CI −1.49 to −0.53; *p* < 0.0001), with low heterogeneity (*I*^2^ = 10%).

Although the magnitude of effect was numerically greater in programmes exceeding 16 weeks compared with shorter-duration interventions, the test for subgroup differences was not statistically significant (*p* = 0.37; *I*^2^ for subgroup difference = 0%). These exploratory analyses therefore do not provide sufficient evidence to conclude that longer-duration interventions are superior to shorter programmes.

## 4 Discussion

The present study quantitatively evaluated the effects of exercise interventions on depressive symptoms in women with PCOS through a systematic synthesis of randomised controlled trials. The pooled analysis demonstrated that exercise significantly reduced depressive symptoms compared with control conditions, with a combined effect size and low overall heterogeneity, although the small number of included studies and limited sample sizes should be considered when interpreting the certainty of the pooled estimate. Baseline assessments confirmed no significant differences in depressive symptom severity between intervention and control groups, supporting the internal validity of subsequent treatment effect estimates. Subgroup analyses were undertaken on an exploratory basis to examine whether intervention characteristics were associated with variation in effect estimates. Resistance training showed a larger pooled effect estimate than aerobic exercise, and the subgroup difference reached statistical significance; however, this finding should still be interpreted cautiously because it was based on a small number of studies. Group-based programmes showed a numerically larger pooled effect estimates compared with individually delivered interventions, although the between-group difference did not reach statistical significance. Similarly, interventions delivered five times per week and those lasting more than 16 weeks demonstrated numerically greater effect sizes than lower-frequency and shorter-duration programmes, respectively; however, these differences were not statistically significant. Collectively, these findings suggest that structured exercise may be a useful non-pharmacological approach for reducing depressive symptoms in women with PCOS, although conclusions regarding optimal intervention characteristics remain tentative.

The findings of this study are consistent with a substantial body of evidence demonstrating the antidepressant effects of exercise (22). Previous research has shown that structured physical activity significantly reduces depressive symptoms in the general population, postpartum women and individuals with chronic metabolic disorders (23, 24). The present analysis extends this evidence base to women with PCOS, suggesting that similar benefits may also be observed in this population. To date, most research in PCOS has focused on metabolic parameters, weight management and reproductive endocrine outcomes (25, 26), with comparatively limited systematic evaluation of psychological morbidity, particularly depressive symptoms. By restricting inclusion to randomised controlled trials and applying quantitative meta-analytic methods, this study addresses an important gap in the literature. An exploratory subgroup analysis suggested that resistance training may be associated with larger reductions in depressive symptoms than aerobic exercise. Although this pattern is of potential interest and is broadly consistent with observations in some chronic disease populations, it should not be regarded as definitive evidence and requires confirmation in larger comparative trials.

The mechanisms through which exercise may alleviate depressive symptoms in women with PCOS are likely multifactorial (27). PCOS is commonly characterised by insulin resistance, chronic low-grade inflammation and endocrine dysregulation, all of which have been implicated in the pathophysiology of depression. Exercise may mitigate depression-related risk by enhancing insulin sensitivity, reducing circulating inflammatory cytokines and improving lipid metabolism, thereby addressing key metabolic and inflammatory pathways linked to mood disturbance. In addition, exercise is known to influence central stress-regulatory systems. Modulation of hypothalamic–pituitary–adrenal (HPA) axis activity may contribute to normalising stress responses, which are frequently dysregulated in depressive states (28). Physical activity has also been associated with increased expression of brain-derived neurotrophic factor (BDNF), supporting neuroplasticity and enhancing emotional regulation (29, 30). Together, these biological effects provide a plausible framework linking exercise to improved psychological outcomes in PCOS. However, these mechanistic explanations are largely inferred from broader literature on exercise and depression or from studies focused on metabolic dysfunction in PCOS, rather than from trials directly designed to test psychological mechanisms in women with PCOS. Therefore, they should be regarded as plausible but still somewhat speculative. The comparatively larger effect observed for resistance training may reflect additional physiological and psychosocial mechanisms. Resistance exercise promotes increases in muscle mass and favourable changes in body composition, which may be particularly salient for women with PCOS who frequently experience body image dissatisfaction due to weight gain, acne and hirsutism. Improvements in physical strength and body composition may enhance self-efficacy and perceived control, contributing to greater psychological benefit. These interacting metabolic and psychosocial pathways likely underpin the multidimensional effects of exercise in this population.

Despite providing a systematic and quantitative synthesis of available evidence, this study has several limitations. First, the number of eligible randomised controlled trials was modest, and the overall sample size was limited. These constraints may affect the precision of pooled effect estimates and reduce the statistical power of subgroup analyses. In particular, comparisons within certain subgroups, such as resistance-based interventions and group-delivered programmes, should be interpreted with caution given the small number of contributing studies. More broadly, the subgroup analyses in this review should be considered exploratory rather than confirmatory. Second, heterogeneity in intervention characteristics—including intensity, duration and delivery format—as well as variation in depression assessment instruments across trials may have introduced methodological variability. Although standardised mean differences were employed to facilitate comparability across measures, residual heterogeneity cannot be excluded. Third, blinding of participants and personnel is inherently challenging in exercise-based interventions. Several included studies presented unclear risk in domains related to blinding and reporting transparency, which may have implications for internal validity. Because depressive symptoms were assessed using self-report or questionnaire-based instruments, lack of blinding may have increased susceptibility to expectation effects or measurement bias, potentially inflating the observed intervention effects. Finally, the limited number of included trials constrains the interpretability of publication bias assessments, and the absence of statistical evidence of bias does not preclude its presence. In addition, although the pooled effect size was statistically significant, the small evidence base means that the practical or clinical significance of this finding should be interpreted cautiously until supported by larger and more methodologically consistent trials.

Future research should prioritise larger, rigorously designed randomised controlled trials with standardised intervention protocols and consistent outcome assessment to better define optimal exercise prescriptions for reducing depressive symptoms in women with PCOS. Head-to-head comparisons of exercise modalities, more precise reporting of intervention dose, and longer follow-up periods would be particularly valuable for clarifying sustainability of effects and clinical applicability.

## 5 Conclusion

This meta-analysis suggests that structured exercise interventions are associated with significant reductions in depressive symptoms among women with polycystic ovary syndrome (PCOS). The pooled findings indicate that exercise may be beneficial; however, the results should be interpreted cautiously in light of the small number of included trials, limited sample sizes, and variation in intervention characteristics. Resistance-based programmes showed a larger pooled effect estimate than aerobic exercise in exploratory subgroup analyses, while higher training frequency and longer intervention duration showed numerically greater improvement, although these differences were not consistently statistically significant.

Taken together, these findings suggest that exercise may represent a useful non-pharmacological approach for addressing the psychological burden associated with PCOS. Given the complex interplay between metabolic dysfunction, endocrine imbalance and mental health in this population, exercise may offer multidimensional therapeutic value. Nevertheless, the limited number of high-quality trials underscores the need for larger, rigorously designed randomised studies to refine optimal exercise prescriptions and to clarify long-term mental health outcomes in women with PCOS.

## Data Availability

Publicly available datasets were analyzed in this study. This data can be found here: because this study is a systematic review and meta-analysis based exclusively on previously published randomised controlled trials, there is no single original dataset deposited in a dedicated repository and therefore no accession number. All data analyzed are publicly available in the respective peer-reviewed journal articles cited in the reference list. The extracted dataset used for meta-analysis is available from the corresponding author upon reasonable request.

## References

[1] Lucassen P Spijker J. Depression. Ned Tijdschr Geneeskd. (2025) 169 39976488

[2] Kennedy SH Eisfeld BS. Clinical aspects of depression. Clin Cornerstone. (1999) 1:1–16. doi: 10.1016/s1098-3597(99)90021-2, 10682174

[3] Dupuis-Lesavre S Mebazaa C Veal C Ribeiro A Boutron I Krause K . Tracking positive emotions in the course of adult depression: A systematic scoping review of longitudinal studies. J Affect Disord. (2026) 393:120387. doi: 10.1016/j.jad.2025.120387, 41076159

[4] Horvath P. Introduction: women, depression, and the struggle for control over the evaluation of self-worth. J Prev Interv Community. (2008) 35:1–3. doi: 10.1300/J005v35n02_01 19842354

[5] Hollon SD. The efficacy and acceptability of psychological interventions for depression: where we are now and where we are going. Epidemiol Psychiatr Sci. (2016) 25:295–300. doi: 10.1017/S2045796015000748, 26310321 PMC7137599

[6] Meier RK. Polycystic ovary syndrome. Nurs Clin North Am. (2018) 53:407–20. doi: 10.1016/j.cnur.2018.04.008, 30100006

[7] Lizneva D Suturina L Walker W Brakta S Gavrilova-Jordan L Azziz R. Criteria, prevalence, and phenotypes of polycystic ovary syndrome. Fertil Steril. (2016) 106:6–15. doi: 10.1016/j.fertnstert.2016.05.003, 27233760

[8] Nandi A Chen Z Patel R Poretsky L. Polycystic ovary syndrome. Endocrinol Metab Clin N Am. (2014) 43:123–47. doi: 10.1016/j.ecl.2013.10.003, 24582095

[9] Cooney LG Dokras A. Depression and anxiety in polycystic ovary syndrome: etiology and treatment. Curr Psychiatry Rep. (2017) 19:83. doi: 10.1007/s11920-017-0834-2, 28929349

[10] Li Y Zhang J Zheng X Lu W Guo J Chen F . Depression, anxiety and self-esteem in adolescent girls with polycystic ovary syndrome: a systematic review and meta-analysis. Front Endocrinol (Lausanne). (2024) 15:1399580. doi: 10.3389/fendo.2024.1399580, 39403587 PMC11471625

[11] Teede HJ Misso ML Costello MF Dokras A Laven J Moran L . Recommendations from the international evidence-based guideline for the assessment and management of polycystic ovary syndrome. Fertil Steril. (2018) 110:364–79. doi: 10.1016/j.fertnstert.2018.05.004, 30033227 PMC6939856

[12] Misso ML Tassone EC Costello MF Dokras A Laven J Moran LJ . Large-scale evidence-based guideline development engaging the international PCOS community. Semin Reprod Med. (2018) 36:028–34. doi: 10.1055/s-0038-1667312, 30189448

[13] Noetel M Sanders T Gallardo-Gómez D Taylor P Del Pozo Cruz B van den Hoek D . Effect of exercise for depression: systematic review and network meta-analysis of randomised controlled trials. BMJ. (2024) 384:e075847. doi: 10.1136/bmj-2023-075847, 38355154 PMC10870815

[14] Kandola A Ashdown-Franks G Hendrikse J Sabiston CM Stubbs B. Physical activity and depression: towards understanding the antidepressant mechanisms of physical activity. Neurosci Biobehav Rev. (2019) 107:525–39. doi: 10.1016/j.neubiorev.2019.09.040, 31586447

[15] Teede HJ Misso ML Costello MF Dokras A Laven J Moran L . Recommendations from the international evidence-based guideline for the assessment and management of polycystic ovary syndrome. Clin Endocrinol. (2018) 89:251–68. doi: 10.1111/cen.13795, 30024653 PMC9052397

[16] Woodward A Klonizakis M Broom D. Exercise and polycystic ovary syndrome. Adv Exp Med Biol. (2020) 1228:123–36. doi: 10.1007/978-981-15-1792-1_8, 32342454

[17] Kite C Lahart IM Afzal I Broom DR Randeva H Kyrou I . Exercise, or exercise and diet for the management of polycystic ovary syndrome: a systematic review and meta-analysis. Syst Rev. (2019) 8:51. doi: 10.1186/s13643-019-0962-3, 30755271 PMC6371542

[18] Kogure GS Lopes IP Ribeiro VB Mendes MC Kodato S Furtado CLM . The effects of aerobic physical exercises on body image among women with polycystic ovary syndrome. J Affect Disord. (2020) 262:350–8. doi: 10.1016/j.jad.2019.11.025, 31735408

[19] Stener-Victorin E Holm G Janson PO Gustafson D Waern M. Acupuncture and physical exercise for affective symptoms and health-related quality of life in polycystic ovary syndrome: secondary analysis from a randomized controlled trial. BMC Complement Altern Med. (2013) 13:131. doi: 10.1186/1472-6882-13-131, 23763822 PMC3684530

[20] Thomson RL Buckley JD Lim SS Noakes M Clifton PM Norman RJ . Lifestyle management improves quality of life and depression in overweight and obese women with polycystic ovary syndrome. Fertil Steril. (2010) 94:1812–6. doi: 10.1016/j.fertnstert.2009.11.001, 20004371

[21] Thomson RL Buckley JD Brinkworth GD. Perceived exercise barriers are reduced and benefits are improved with lifestyle modification in overweight and obese women with polycystic ovary syndrome: a randomised controlled trial. BMC Womens Health. (2016) 16:14. doi: 10.1186/s12905-016-0292-8, 26960762 PMC4784413

[22] Kaushik A Wasir AS Pillai AA Singla A A LR Goyal N. Effect of high-intensity interval training and moderate-intensity continuous training on the cardiometabolic profile and psychological outcomes in patients with polycystic ovary syndrome: a systematic review and META analysis. J Am Coll Cardiol. (2025) 85:2626. doi: 10.1016/s0735-1097(25)03110-9

[23] Coll CVN Domingues MR Stein A da Silva BGC Bassani DG Hartwig FP . Efficacy of regular exercise during pregnancy on the prevention of postpartum depression: the PAMELA randomized clinical trial. JAMA Netw Open. (2019) 2:e186861. doi: 10.1001/jamanetworkopen.2018.6861, 30646198 PMC6324311

[24] Pedersen BK Saltin B. Exercise as medicine - evidence for prescribing exercise as therapy in 26 different chronic diseases. Scand J Med Sci Sports. (2015) 25:1–72. doi: 10.1111/sms.12581, 26606383

[25] Ruiz-González D Cavero-Redondo I Hernández-Martínez A Baena-Raya A Martínez-Forte S Altmäe S . Comparative efficacy of exercise, diet and/or pharmacological interventions on BMI, ovulation, and hormonal profile in reproductive-aged women with overweight or obesity: a systematic review and network meta-analysis. Hum Reprod Update. (2024) 30:472–87. doi: 10.1093/humupd/dmae008, 38627233 PMC11215161

[26] Cowan S Lim S Alycia C Pirotta S Thomson R Gibson-Helm M . Lifestyle management in polycystic ovary syndrome - beyond diet and physical activity. BMC Endocr Disord. (2023) 23:14. doi: 10.1186/s12902-022-01208-y, 36647089 PMC9841505

[27] Li S Jia S Yun S Guo Z Wang X Zhang Q. The relationship between different components and levels of physical exercise, depressive symptoms, inhibitory control, and possible cognitive neural mechanisms in college students. CNS Neurosci Ther. (2025) 31:e70520. doi: 10.1111/cns.70520, 40745688 PMC12313544

[28] Archer T Josefsson T Lindwall M. Effects of physical exercise on depressive symptoms and biomarkers in depression. CNS Neurol Disord Drug Targets. (2014) 13:1640–53. doi: 10.2174/1871527313666141130203245, 25470398

[29] Salehi I Hosseini SM Haghighi M Jahangard L Bajoghli H Gerber M . Electroconvulsive therapy (ECT) and aerobic exercise training (AET) increased plasma BDNF and ameliorated depressive symptoms in patients suffering from major depressive disorder. J Psychiatr Res. (2016) 76:1–8. doi: 10.1016/j.jpsychires.2016.01.012, 26859236

[30] Moradi-Kor N Dadkhah M Ghanbari A Rashidipour H Bandegi AR Barati M . Protective effects of spirulina platensis, voluntary exercise and environmental interventions against adolescent stress-induced anxiety and depressive-like symptoms, oxidative stress and alterations of BDNF and 5HT-3 receptors of the prefrontal cortex in female rats. Neuropsychiatr Dis Treat. (2020) 16:1777–94. doi: 10.2147/NDT.S247599, 32801713 PMC7387863

