# Supplementary material for: Exercise-based interventions for depression in women with polycystic ovary syndrome: a systematic review and meta-analysis
Source: Front Public Health. 2026 Apr 13;14:1802184. doi: 10.3389/fpubh.2026.1802184 (PMC13111113; doi:10.3389/fpubh.2026.1802184)
Supplement: Supplementary file 1 [file Data_sheet_1.pdf]

	Random sequence generation (selection bias)	Allocation concealment (selection bias)	Blinding of participants and personnel (performance bias)	Blinding of outcome assessment (detection bias)	Incomplete outcome data (attrition bias)	Selective reporting (reporting bias)	Other bias
Kogure, G. S(1) 2020	+	+	−	?	+	+	?
Kogure, G. S(2) 2020							
Lopes, I P (1) 2018	+	+	−	?	+	+	+
Lopes, I P (2) 2018							
Santos, I. K 2022	+	+	+	−	+	?	?
Stener-Victorin, E 2013	+	?	−	?	+	+	?
Thomson, R. L(1) 2010	+	?	−	?	+	?	?
Thomson, R. L(2) 2010							
Thomson, R. L 2016	+	+	−	−	+	+	?
Vizza, L 2016	+	+	−	?	+	+	?
