# Supplementary material for: Exercise-based interventions for depression in women with polycystic ovary syndrome: a systematic review and meta-analysis
Source: Front Public Health. 2026 Apr 13;14:1802184. doi: 10.3389/fpubh.2026.1802184 (PMC13111113; doi:10.3389/fpubh.2026.1802184)
Supplement: Supplementary file 2 [file Table_1.docx]

**Pubmed search algorithm**

#1 "Depression"[Mesh Terms]

#2 "depressive symptoms"[Title/Abstract] OR "depressive symptom"[Title/Abstract] OR "symptom depressive"[Title/Abstract] OR "emotional depression"[Title/Abstract] OR "depression emotional"[Title/Abstract]

#3 #1 OR #2

#4 "Exercise"[Mesh Terms]

#5 "Exercises"[Title/Abstract] OR "physical activity"[Title/Abstract] OR "activities physical"[Title/Abstract] OR "activity physical"[Title/Abstract] OR "physical activities"[Title/Abstract] OR "exercise physical"[Title/Abstract] OR "exercises physical"[Title/Abstract] OR "physical exercise"[Title/Abstract] OR "physical exercises"[Title/Abstract] OR "acute exercise"[Title/Abstract] OR "acute exercises"[Title/Abstract] OR "exercise acute"[Title/Abstract] OR "exercises acute"[Title/Abstract] OR "exercise isometric"[Title/Abstract] OR "exercises isometric"[Title/Abstract] OR "isometric exercises"[Title/Abstract] OR "isometric exercise"[Title/Abstract] OR "exercise aerobic"[Title/Abstract] OR "aerobic exercise"[Title/Abstract] OR "aerobic exercises"[Title/Abstract] OR "exercises aerobic"[Title/Abstract] OR "exercise training"[Title/Abstract] OR "exercise trainings"[Title/Abstract] OR "training exercise"[Title/Abstract] OR (("education"[MeSH Subheading] OR "education"[All Fields] OR "Training"[All Fields] OR "education"[MeSH Terms] OR "train"[All Fields] OR "train s"[All Fields] OR "trained"[All Fields] OR "training s"[All Fields] OR "Trainings"[All Fields] OR "trains"[All Fields]) AND "Exercise"[Title/Abstract])

#6 #4 OR #5

#7 "Polycystic Ovary Syndrome"[Mesh Terms]

#8 "Ovary Syndrome, Polycystic"[Title/Abstract] OR "Syndrome, Polycystic Ovary"[Title/Abstract] OR "Polycystic Ovarian Syndrome"[Title/Abstract] OR "Ovarian Syndrome, Polycystic"[Title/Abstract] OR "Polycystic Ovary Syndrome 1"[Title/Abstract] OR "Sclerocystic Ovarian Degeneration"[Title/Abstract] OR " Sclerocystic Ovary Syndrome"[Title/Abstract] OR " Stein-Leventhal Syndrome"[Title/Abstract] OR " Stein Leventhal Syndrome"[Title/Abstract] OR " Syndrome, Stein-Leventhal"[Title/Abstract] OR " Sclerocystic Ovaries"[Title/Abstract] OR " Sclerocystic Ovary"[Title/Abstract]

#9 #7 OR #8

#10 "randomized controlled trial"[Publication Type] OR "randomized"[Title/Abstract] OR "placebo"[Title/Abstract]

#11 #3 AND #6 AND #9 AND #10

**Web of Science search algorithm**

#1 TS=(Depression OR Depressive Symptoms OR Depressive Symptom OR Symptom, Depressive OR Emotional Depression OR Depression, Emotional)

#2 TS=(Exercise, Acute OR Exercises, Acute OR Exercise, Isometric OR Exercises, Isometric OR Isometric Exercises OR Isometric Exercise OR Exercise, Aerobic OR Aerobic Exercise OR Aerobic Exercises OR Exercises, Aerobic OR Exercise Training OR Exercise Trainings OR Training, Exercise OR Trainings, Exercise) and Preprint Citation Index (Exclude – Database)

#3 TS=(Polycystic Ovary Syndrome OR Ovary Syndrome, Polycystic OR Syndrome, Polycystic Ovary OR Polycystic Ovarian Syndrome OR Ovarian Syndrome, Polycystic OR Polycystic Ovary Syndrome 1 OR Sclerocystic Ovarian Degeneration OR Sclerocystic Ovary Syndrome OR Sclerocystic Ovary Syndrome OR Stein-Leventhal Syndrome OR Stein Leventhal Syndrome OR Syndrome, Stein-Leventhal OR Sclerocystic Ovaries OR Sclerocystic Ovary)

#4 TS=(randomized controlled trial OR randomized OR placebo OR RCT )

#5 #1 AND #2 AND #3 AND #4

**Embase search algorithm**

#1 'depression'/exp OR depression

#2 'depressive symptoms':ab,ti OR 'depressive symptom':ab,ti OR 'symptom, depressive':ab,ti OR 'emotional depression':ab,ti OR 'depression, emotional':ab,ti

#3 #1 OR #2

#4 exercise

#5 'exercises':ab,ti OR 'physical activity':ab,ti OR 'activities, physical':ab,ti OR 'activity, physical':ab,ti OR 'physical activities':ab,ti OR 'exercise, physical':ab,ti OR 'exercises, physical':ab,ti OR 'physical exercise':ab,ti OR 'physical exercises':ab,ti OR 'acute exercise':ab,ti OR 'acute exercises':ab,ti OR 'exercise, acute':ab,ti OR 'exercises, acute':ab,ti OR 'exercise, isometric':ab,ti OR 'exercises, isometric':ab,ti OR 'isometric exercises':ab,ti OR 'isometric exercise':ab,ti OR 'exercise, aerobic':ab,ti OR 'aerobic exercise':ab,ti OR 'aerobic exercises':ab,ti OR 'exercises, aerobic':ab,ti OR 'exercise training':ab,ti OR 'exercise trainings':ab,ti OR 'training, exercise':ab,ti OR 'trainings, exercise':ab,ti

#6 #4 OR #5

#7 'Polycystic Ovary Syndrome':ab,ti OR 'Ovary Syndrome, Polycystic':ab,ti OR 'Syndrome, Polycystic Ovary':ab,ti OR 'Polycystic Ovarian Syndrome':ab,ti OR 'Ovarian Syndrome, Polycystic':ab,ti OR 'Polycystic Ovary Syndrome 1':ab,ti OR 'Ovarian Degeneration':ab,ti OR 'Sclerocystic Ovary Syndrome':ab,ti OR 'Sclerocystic Ovary Syndrome':ab,ti OR 'Stein-Leventhal Syndrome':ab,ti OR 'Stein Leventhal Syndrome':ab,ti OR 'Syndrome, Stein-Leventhal':ab,ti OR 'Sclerocystic Ovaries':ab,ti OR 'Sclerocystic Ovary':ab,ti

#8 'randomized controlled trial':ab,ti OR 'randomized':ab,ti OR 'placebo':ab,ti OR 'rct':ab,ti

#9 #3 AND #6 AND #7 AND #8

**Cochrane Library search algorithm**

#1 Depression

#2 (Depressive Symptoms):ab,ti,kw OR (Depressive Symptom):ab,ti,kw OR (Symptom, Depressive):ab,ti,kw OR (Emotional Depression):ab,ti,kw OR (Depression, Emotional):ab,ti,kw

#3 #1 OR #2

#4 exercise

#5 (Exercises):ab,ti,kw OR (Physical Activity):ab,ti,kw OR (Activities, Physical):ab,ti,kw OR (Activity, Physical):ab,ti,kw OR (Physical Activities):ab,ti,kw OR (Exercise, Physical):ab,ti,kw OR (Exercises, Physical):ab,ti,kw OR (Physical Exercise):ab,ti,kw OR (Physical Exercises):ab,ti,kw OR (Acute Exercise):ab,ti,kw OR (Acute Exercises):ab,ti,kw OR (Exercise, Acute):ab,ti,kw OR (Exercises, Acute):ab,ti,kw OR (Exercise, Isometric):ab,ti,kw OR (Exercises, Isometric):ab,ti,kw OR (Isometric Exercises):ab,ti,kw OR (Isometric Exercise):ab,ti,kw OR (Exercise, Aerobic):ab,ti,kw OR (Aerobic Exercise):ab,ti,kw OR (Aerobic Exercises):ab,ti,kw

#6 #4 OR #5

#7 (Polycystic Ovary Syndrome):ab,ti,kw OR (Ovary Syndrome, Polycystic):ab,ti,kw OR (Syndrome, Polycystic Ovary):ab,ti,kw OR (Polycystic Ovarian Syndrome):ab,ti,kw OR (Ovarian Syndrome, Polycystic):ab,ti,kw OR (Polycystic Ovary Syndrome 1):ab,ti,kw OR (Ovarian Degeneration):ab,ti,kw OR (Sclerocystic Ovary Syndrome):ab,ti,kw OR (Sclerocystic Ovary Syndrome):ab,ti,kw OR (Stein-Leventhal Syndrome):ab,ti,kw OR (Stein Leventhal Syndrome):ab,ti,kw OR (Syndrome, Stein-Leventhal):ab,ti,kw OR (Sclerocystic Ovaries):ab,ti,kw OR (Sclerocystic Ovary):ab,ti,kw

#8 (randomized controlled trial):ab,ti,kw OR (randomized):ab,ti,kw OR (placebo):ab,ti,kw OR (RCT):ab,ti,kw

#9 #3 AND #6 AND #7 AND #8
